# PHD-finger domain protein 5A functions as a novel oncoprotein in lung adenocarcinoma

**DOI:** 10.1186/s13046-018-0736-0

**Published:** 2018-03-22

**Authors:** Yan Yang, Jian Zhu, Tiantian Zhang, Jing Liu, Yumei Li, Yue Zhu, Lingjie Xu, Rui Wang, Fang Su, Yurong Ou, Qiong Wu

**Affiliations:** 1 Department of Medical Oncology, The First Affiliated Hospital of Bengbu Medical College, Bengbu, 233004 People’s Republic of China; 2 Department of Cardiology, The First Affiliated Hospital of Bengbu Medical College, Bengbu, 233004 People’s Republic of China; 3 Department of Pathology, The First Affiliated Hospital of Bengbu Medical College, Bengbu, 233004 Anhui People’s Republic of China

**Keywords:** Lung adenocarcinoma, PHF5A, Prognostic biomarker, Proliferation, Tumor invasion

## Abstract

**Background:**

PHD-finger domain protein 5A (PHF5A) is a highly conserved small transcriptional regulator also involved in pre-mRNA splicing; however, its biological functions and molecular mechanisms in non-small cell lung cancer (NSCLC) have not yet been investigated. The purpose of this study was to determine the functional relevance and therapeutic potential of PHF5A in lung adenocarcinoma (LAC).

**Methods:**

The expression of PHF5A in LAC tissues and adjacent non-tumor (ANT) tissues was investigated using immunohistochemistry of a tissue microarray, qRT-PCR, western blot and bioinformatics. The function of PHF5A was determined using several in vitro assays and also in vivo assay by lentiviral vector-mediated PHF5A depletion in LAC cell lines.

**Results:**

PHF5A was highly upregulated in LAC tissues compared with the ANT counterparts, and closely associated with tumor progression and poor patient prognosis. These results were further confirmed by findings of the TCGA database. Moreover, functional studies demonstrated that PHF5A knockdown not only resulted in reduced cell proliferation, increased cell apoptosis, and cell cycle arrest, but also suppressed migration and invasion in LAC cells. PHF5A silencing was also found to inhibit LAC tumor growth in nude mice. Microarray and bioinformatics analyses revealed that PHF5A depletion led to dysregulation of multiple tumor signaling pathways; selected factors in key signaling pathways were verified in vitro.

**Conclusions:**

The data suggest for the first time that PHF5A is an oncoprotein that contributes to LAC progression by regulating multiple signaling pathways, and may constitute a prognostic factor and potential new therapeutic target in NSCLC.

**Electronic supplementary material:**

The online version of this article (10.1186/s13046-018-0736-0) contains supplementary material, which is available to authorized users.

## Background

Lung cancer is the most common malignancy worldwide, with morbidity and mortality ranking first among all cancers [1]. About 80% to 85% of clinical lung cancer cases are non-small cell lung cancer (NSCLC), with adenocarcinoma being the most common histological type [2]. For locally advanced, recurrent, or metastatic NSCLC that cannot be fully resected, current major therapeutic strategies include palliative chemotherapy and targeted therapy combined with or without radiotherapy. However, prognosis in such patients is generally not satisfactory, with an overall 5-year survival rate still hovering around 15% [3]. Therefore, it is urgent to explore the mechanisms that regulate tumor pathogenesis and identify novel potential therapeutic targets.

PHD-finger domain protein 5a (*Phf5a*), a member of the superfamily of PHD-finger genes, encodes a protein of 110 amino acids with the PHD zinc finger domain [4]. The PHF5A protein is expressed ubiquitously in the nucleus of eukaryotes from yeasts to humans, in a highly conserved manner during evolution. PHF5A in rats acts as an important small transcription factor or cofactor, through binding to the promoter of the *connexin43* gene, to increase its expression in response to estrogen induction [5]. Subsequently, PHF5A is characterized as an important component of the splicing factor SF3b complex [6], thereby directly participating in protein-protein interactions or regulating downstream genes through the RNA splicing pathway [6–8]. Previous studies have shown that PHF5A not only plays an important role in the processes of chromatin remodeling [4, 9], morphological development of tissues and organs [9], and maintenance of stem cell pluripotency [10, 11], but is also involved in the regulation of the cell cycle [12] as well as cell growth and differentiation [4, 11, 13].

Assessing the association of PHF5A with tumors, Falck et al. [14] found that PHF5A expression in endometrial adenocarcinoma was increased compared with that of benign samples. In addition, Hubert et al. [13] demonstrated a novel requirement for PHF5A in glioblastoma stem cell initiation and maintenance, by showing that PHF5A knockdown disrupted splicing of multiple essential genes and induced cell cycle arrest and loss of viability. These findings suggested that PHF5A could play a role in tumor development as a general transcription regulator for different genes. However, the molecular and biological functions of *Phf5a* in lung cancer, particularly lung adenocarcinoma (LAC), remain unknown. This study, for the first time, assessed the role and molecular mechanism of *Phf5a* in LAC cell proliferation, apoptosis, and invasion. Our findings are expected to reveal novel biomarkers and therapeutic targets, providing a new avenue for the treatment of NSCLC.

## Methods

### Clinical samples

A total of 70 pairs of primary lung cancer and the corresponding adjacent non-tumor (ANT) samples were collected from patients undergoing surgical resection in the First Affiliated Hospital of Bengbu Medical College (Bengbu, China), between January 2012 and June 2013. The patients received no treatment preoperatively, and were confirmed to have lung adenocarcinoma (LAC) pathologically. Detailed clinicopathological data were recorded, including patient′s age and gender, tumor size, tumor histological grade, lymph node metastasis and clinical stage. Tumor histological grade assessments were based on the 2011 IASLC/ATS/ERS multidisciplinary classification of LAC. Tumor clinical stages were classified according to the 7th edition of the AJCC cancer staging manual [15]. Three additional pairs of matched LAC/ANT lung tissue samples for qRT-qPCR, Western blot, and IHC were obtained from surgical patients in October 2017 in our institution. ANT lung tissues were taken from the tissue ≥5 cm away from the tumor in LAC patients. Approval was obtained from the medical ethics committee of our institute, and written informed consent was provided by all patients. The specimens were immediately snap frozen in liquid nitrogen and stored at − 80 °C until use.

### LAC tissue microarray (TMA) construction and immunohistochemistry (IHC)

LAC TMAs containing 70 pairs of matched LAC/ANT lung samples were constructed at Shanghai Outdo Biotech Co., Ltd (Shanghai, China). Rabbit polyclonal anti-human PHF5A antibodies (1:50, Proteintech, China) were used for immunohistochemistry according to a two-step protocol. PHF5A staining was detected mainly in the nucleus. The intensity of positive signals was scored as: 1, negative (no staining); 2, weak (light yellow); 3, moderate (yellowish brown); 4, strong (brown). The extent of positivity was scored based on the percentage of positive cells: 0, <5%; 1, 5%~ 25%; 2, 26%~ 50%; 3, 51%~ 75%; 4, >75%. The staining index (SI) was determined as the final score by multiplying the above scores, yielding a range from 0 to 16. Then, the median SI value of 8 was selected as cut off, and samples with SI ≥ 8 and SI < 8 were assigned to the high and low expression groups, respectively.

### Cell lines and cell culture

The human LAC cell lines H1299 and H1975 were obtained from the Chinese Academy of Sciences (Shanghai, China), and authenticated using short tandem repeat (STR) loci by Shanghai GeneChem Co., Ltd. (Shanghai, China). Cells were cultured in RPMI 1640 (Thermo Fisher Scientific, USA) supplemented with 10% fetal bovine serum (FBS, HyClone, USA), 100 U/ml penicillin (Gibco, USA), and 100 μg/ml streptomycin (Gibco, USA) at 37 °C in a humidified atmosphere containing 5% CO_2_.

### shRNA cloning and lentiviral transfection

Lentiviral vectors were purchased from Shanghai GeneChem Co., Ltd. (Shanghai, China). The short hairpin RNA (shRNA) sequence targeting PHF5A (shPHF5A) was 5′- ATCGGAAGACTGTGTGAAA -3′, as confirmed by sequencing. A non-silencing shRNA sequence was used as the negative control (NC) (target sequence 5′- TTCTCCGAACGTGTCACGT -3′) (shCtrl). The cells were seeded into a 6-well plate (~ 5 × 10^4^ cells per well) and incubated at 37 °C with 5% CO_2_ until ~ 40% confluence before transfection. Lipofectamine™ 2000 (Invitrogen, USA) was used for transfection, strictly according to the manufacturer′s instructions.

### Western blot and qRT-PCR

Western blot and qRT-PCR were performed as described in our previous study [16]. Primary antibodies for Western blot were: PHF5A (1:500; Invitrogen); IGFBP3 (1:500; Abcam); PIK3CB (1:500; CST); AKT2 (1:500; Abcam); DDIT3 (1:200; Abcam); Skp2 (1:500; Abcam); P53 (1:1000; CST); GAPDH (1:2000; Santa Cruz Biotechnology). The primers used for qRT-PCR are listed in Additional file 1: Table S1. Gene and protein expression levels were normalized to those of the internal control GAPDH.

### Plate analysis with the adherent cell cytometry system Celigo™

This assay for rapid quantification of cellular fluorescence was performed as described previously [17]. In brief, the transfected cells were trypsin-digested, resuspended, and seeded into 96-well plates at a density of 2000 cells/well for 5 consecutive days. Plates were analyzed on a Celigo image cytometer (Nexcelom, USA), equipped with bright field and fluorescent channels. The green fluorescence tagged shRNA GFP was used to quantify cellular shRNA uptake.

### Colony formation assays

In the logarithmic growth phase, H1299 and H1975 cells were trypsinized, counted, seeded into 6-well plates at 600 cells/well, and cultured for 10~ 14 days at 37 °C in 5% CO_2_. After three washes with PBS, the cells were fixed with methanol and stained with 0.1% crystal violet. The colonies were then washed, photographed, and counted.

### Apoptosis assays

Apoptosis was assessed by Annexin V-based flow cytometry as we previously described [18] with slight modifications. Briefly, transfected cells were harvested, washed with cold PBS, and resuspended in 200 μl binding buffer containing 10 μl Annexin V-APC (eBioscience, USA). After incubation in the dark for 10 min at room temperature, the stained cells were analyzed by flow cytometry (Millipore, USA).

### Cell cycle assays

Lentivirus-transfected cells cultured in 6-cm dishes were cultured to 80% confluence, trypsinized, washed, and fixed with 70% ice-cold ethanol at 4 °C for 1 h. Then, the fixed cells were treated with ribonuclease (Fermentas, USA) for 20 min at 37 °C, and stained with 40 μg/ml propidium iodide (PI) (Sigma-Aldrich, USA). Cellular DNA content was determined by quantitative flow cytometry on a FACSCalibur flow cytometer (BD Biosciences, USA). The percentages of cells in different growth phases (G0/G1, S and G2/M) were analyzed by the CellQuest software (BD Biosciences, USA).

### Animal studies, H&E staining, and IHC

All animal experiments were performed according to institutional guidelines. For xenograft assays, H1299 cells (1 × 10^7^) were resuspended in 200 μl serum-free RPMI 1640 and Matrigel (BD Biosciences; 1:1), and implanted subcutaneously into the flanks of 4-week old BALB/c nu/nu female nude mice. The mice were monitored every 3 days; tumor length and width measurements were performed with calipers, and tumor volumes were derived as length×width^2^ × 0.5 (mm^3^). At 30 days, tumors were detected by an IVIS imaging system, excised, weighted, and paraffin-embedded following necropsy. Serial 5.0 μm sections were obtained and assessed by IHC using anti-PHF5A and anti-Ki67 antibodies (Proteintech). The proliferation index was determined as the proportion of Ki67-positive cells.

### Wound-healing and transwell invasion assays

Wound-healing and transwell invasion assays were performed to determine the migration and invasion capabilities of tumor cells, respectively, as described previously [16].

### Microarray gene expression and bioinformatics analysis

Total RNA from H1299 cells after transfection with control or PHF5A-targeting shRNAs was isolated with TRIzol Reagent (Invitrogen, Carlsbad, CA, USA). Then, NanoDrop 2000 (Thermo Fisher Scientific Inc., DE, USA) and Agilent Bioanalyzer 2100 (Agilent Technologies Inc., Santa Clara, CA, USA) were used to assess RNA integrity. Biotin-labeled amplified RNA (aRNA) was generated with GeneChip 3′IVT Express Kit (Affymetrix Inc., Santa Clara, CA, USA) and purified. After fragmentation, the aRNA was hybridized onto Affymetrix GeneChip 133 Plus 2.0 Arrays (Affymetrix Inc., Santa Clara, CA, USA). The chips were then stained with phycoerythrin and washed on a GeneChip Fluidics Station 450. Microarray signals were scanned on an Affymetrix GeneChip Scanner 3000 and analyzed with the Affymetrix GeneChip Command Console™ 1.1 software. Finally, the image signals were transformed into digital information and analyzed with the SAM software. Ingenuity pathway analysis (IPA) was performed for the tentative exploration of protein networks of PHF5A in lung cancer cells with the IPA Software (Ingenuity Systems, Redwood City, CA, USA). Differentially expressed genes between the shPHF5A and shCtrl groups with corrected *p* < 0.05 and |Z-score| > 2.0 were considered to be significantly differentially expressed.

### Statistical analysis

Statistical tests for data analysis included the Chi-square test, Fisher’s exact test, Mann-Whitney *U* test, Spearman’s correlation test, Log-rank test, Gehan-Breslow-Wilcoxon test, and paired/unpaired Student’s *t* tests. Data represent mean ± SD. *P* < 0.05 was considered statistically significant.

## Results

### PHF5A overexpression is correlated with LAC progression and poor prognosis

**Fig. 1:**
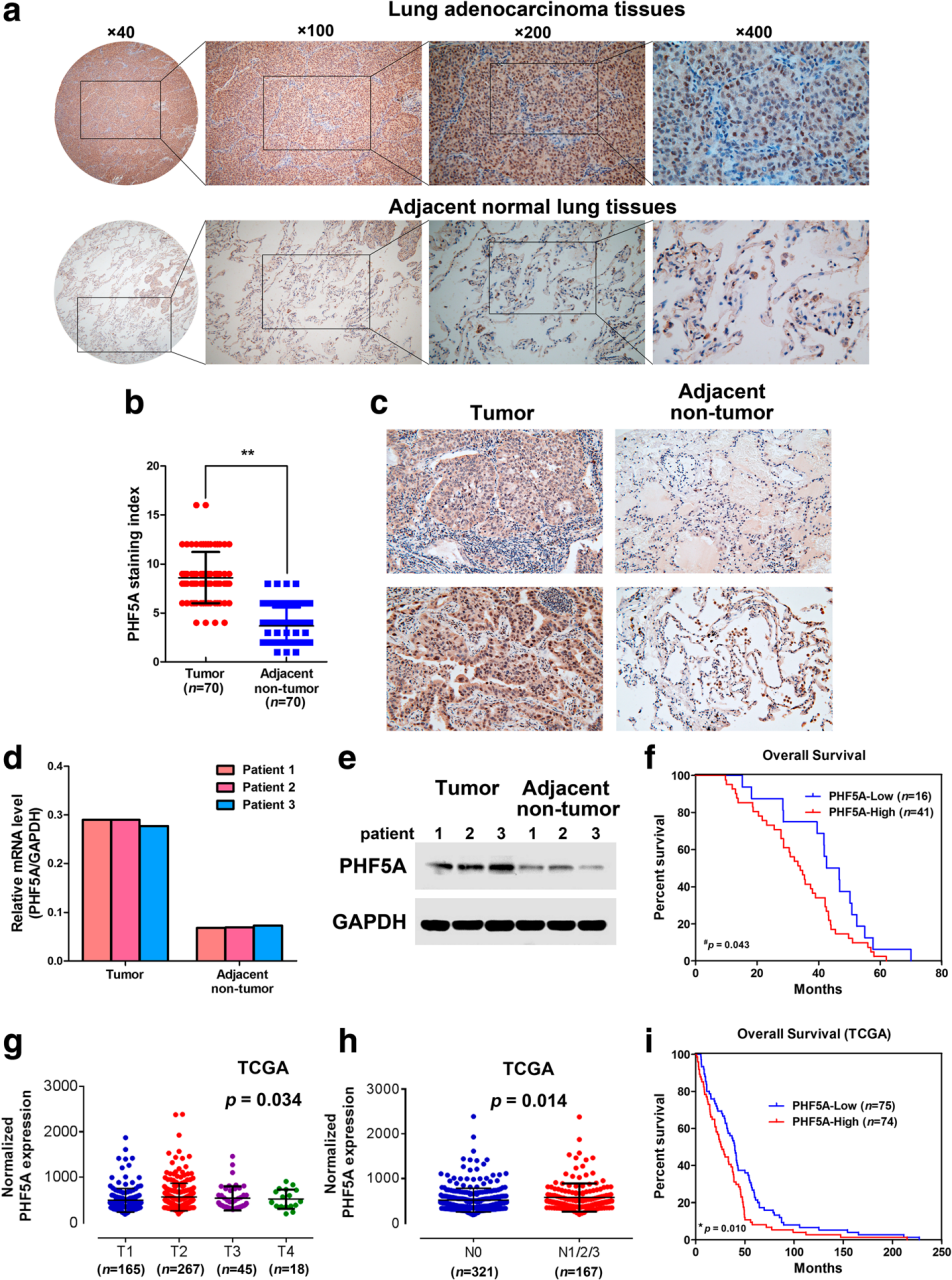
PHF5A overexpression is associated with lung adenocarcinoma (LAC) progression and poor prognosis. **a** PHF5A expression in LAC tissue microarrays. Representative images of LAC tissue samples with strong nuclear staining (brown), with adjacent noncancerous normal lung tissues showing weakly positive PHF5A staining. **b** The staining index (SI) of PHF5A expression in LAC tissues was significantly higher than that of normal paired samples. **c-e** PHF5A expression was confirmed in three fresh paired primary LAC tissues and matched adjacent non-tumor tissues from the same patient, by IHC (**c**), qRT-PCR (**d**) and Western blot (**e**). **f** Kaplan-Meier survival curves for LAC patients with high and low PHF5A expression levels, respectively. LAC patients with high PHF5A expression showed poorer survival compared with the low PHF5A group (*n* = 57; *P* = 0.043, ^#^ compared by Gehan-Breslow-Wilcoxon test). **g-h** TCGA data indicated that *Phf5a* mRNA levels in LAC tissues were associated with T stage (**g**) and N stage (**h**). **i** Kaplan-Meier survival curves comparing LAC patients with low and high *Phf5a* expression levels (*n* = 149; *P* = 0.010, ^★^compared by the log-rank test; TCGA). High and low expression levels were based on the median value of *Phf5a* mRNA. ^**^P < 0.01

We first designed and screened a panel of TMAs to assess PHF5A expression in LAC, and found that the protein was mainly localized in the nucleus (Fig. 1a). Different staining intensities for PHF5A were shown in Additional file 2: Figure S1. PHF5A was highly expressed in up to 72.9% (51/70) LAC samples but showed significantly lower levels in ANT normal lung tissues (Fig. 1b). IHC, qRT-PCR, and Western blot were performed in three fresh paired LAC/ANT lung tissues to further confirm the expression pattern of PHF5A in human LAC. Comparative analysis revealed that PHF5A was markedly overexpressed in primary LAC samples compared with the matched ANT tissue specimens (Fig. 1c-e). Clinicopathological analysis indicated that PHF5A expression was not associated with patient’ age or gender, or histological grade, but positively correlated with tumor size (*P* = 0.015), lymph node metastasis (*P* = 0.002), and clinical stage (*P* = 0.003) (Table 1). Survival analysis suggested that LAC patients with high PHF5A expression had poor prognosis (Fig. 1f).

**Table 1:** Relationship between PHF5A expression and clinicopathological parameters of 70 lung adenocarcinoma samples

Variable	*n*	PHF5A expression		*χ*^*2*^ value	*p* value
		low	high		
Age (years)					
< 60	38	13	25	2.100	0.183
≥ 60	32	6	26		
Gender					
Male	36	11	25	0.437	0.596
Female	34	8	26		
Tumor size (cm)					
≤ 3	20	10	10	7.397	0.015^a^
> 3	50	9	41		
Histological grade					
Low-intermediate	48	15	33	1.303	0.386
High	22	4	18		
Lymph node metastasis					
Negative	26	13	13	10.928	0.002^a^
Positive	44	6	38		
TNM stage					
I–II	53	19	34	6.650	0.003^a^
III–IV	17	0	17		

^a^Significantly different

Analysis of RNA-sequencing datasets of paired LAC/ANT lung tissues from The Cancer Genome Atlas (TCGA) demonstrated that *Phf5a* expression was correlated with T stage (*P* = 0.034) and N stage (*P* = 0.014) in patients with LAC (Fig. 1g-h, Additional file 3: Tables S2 and Additional file 4: Table S3). Kaplan-Meier survival analysis of TCGA data also revealed that higher expression of *Phf5a* was correlated with shorter overall survival (Fig. 1i). Collectively, these findings consistently suggested that PHF5A overexpression might be related to tumor development, and could serve as a new diagnostic and prognostic indicator in LAC.

### PHF5A knockdown inhibits cell growth in vitro

**Fig. 2:**
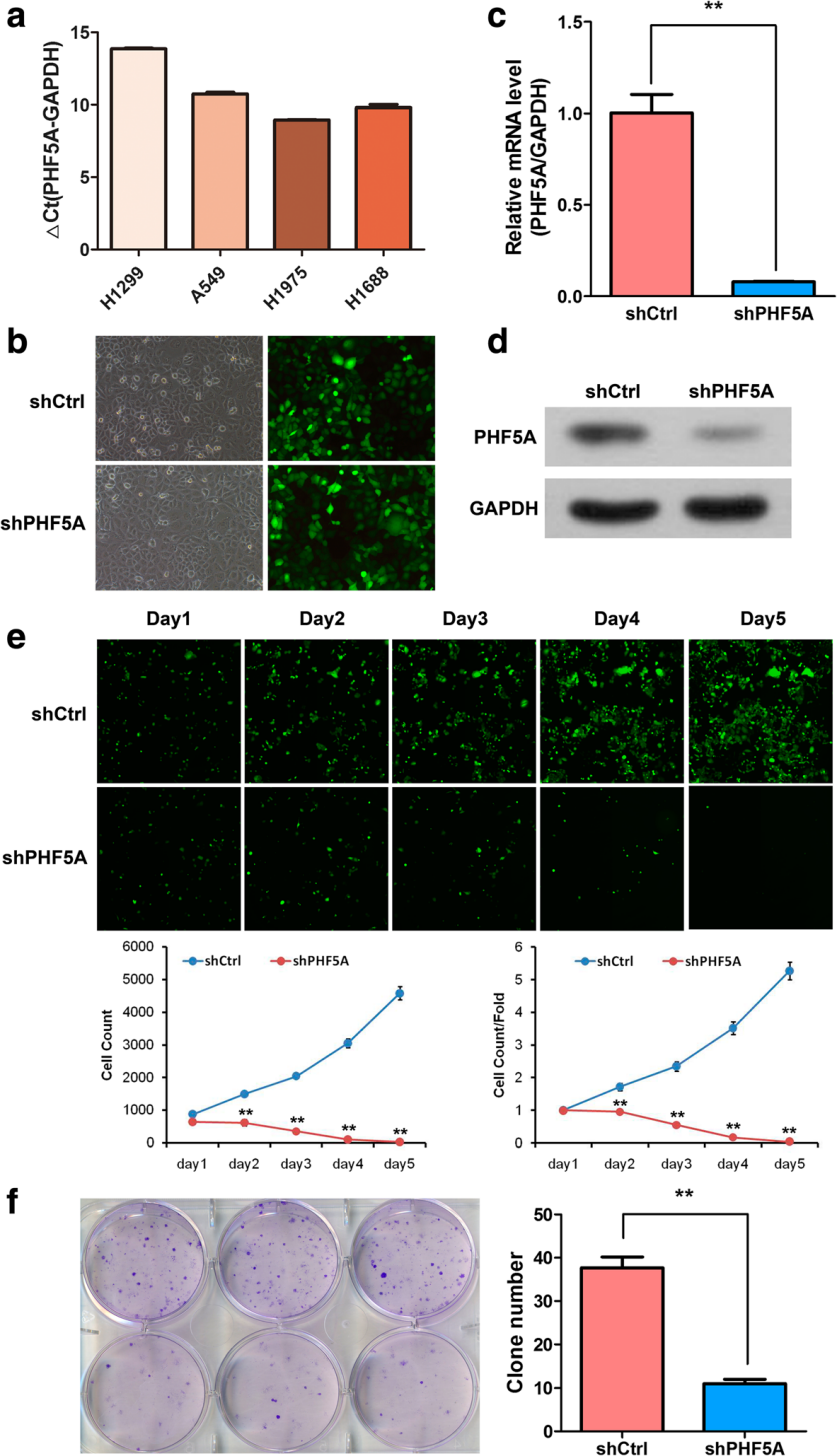
Effect of PHF5A knockdown on cell proliferation and colony formation in H1299 cells. **a** *Phf5a* mRNA levels from four common LAC cell lines were assessed by qRT-PCR. **b-d** Lentivirus-mediated shPHF5A knockdown in H1299 cells was performed via lentiviral infection (**b**), and qRT-PCR (**c**) and Western blot (**d**) were used to assess silencing efficacy. **e** Cellomics assay images and fluorescence quantification over five days in shCtrl and shPHF5A-transfected H1299 cells. **f** Colony formation assay was used to evaluate H1299 cell growth after PHF5A knockdown. ^**^P < 0.01; ^**^compared with the shCtrl group (**e**)

To assess the biological function of PHF5A in vitro, *Phf5a* mRNA expression was assessed in a panel of LAC cell lines (H1299, A549, H1975, and H1688) by qRT-PCR (Fig. 2a). Finally, the H1299 cell line was selected for subsequent studies, since it expressed moderate amounts of endogenous *Phf5a*. The interfering lentiviral vector targeting *Phf5a* was successfully transfected into H1299 cells (Fig. 2b), and significantly inhibited *Phf5a* expression at the gene (Fig. 2c) and protein (Fig. 2d) levels, indicating that the model was successfully established.

**Fig. 3:**
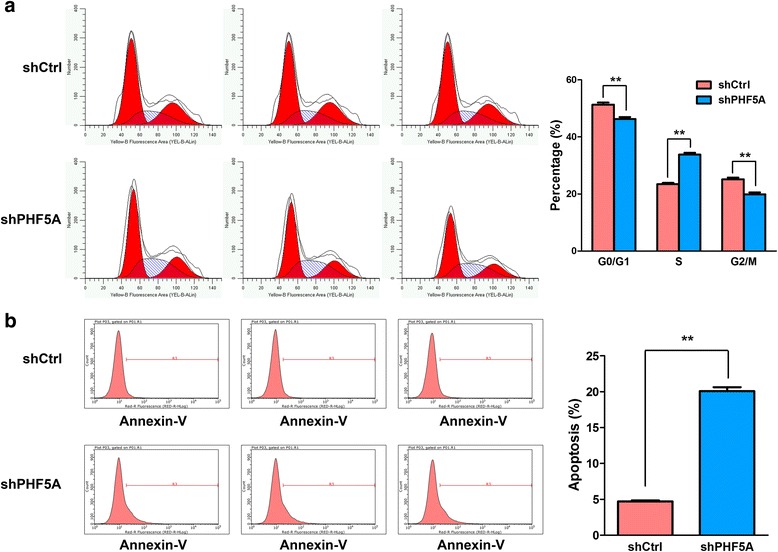
Effect of PHF5A knockdown on cell cycle progression and apoptosis in H1299 cells. **a** Cell cycle was assessed in H1299 cells by flow cytometry five days after transfection with the indicated shRNAs. Representative flow-cytograms are shown, as well as diagrams quantifying cell fractions in the G_0_/G_1_, S, and G_2_/M phases. **b** Apoptosis was evaluated by flow cytometry in PHF5A knockdown and control H1299 cells. Representative flow-cytograms are shown, and apoptotic rates were derived as the percentages of Annexin V-APC positive cells. ^**^P < 0.01

Consequently, PHF5A knockdown in H1299 suppressed cell growth as determined by Cellomics assay during a five-day culture (Fig. 2e). Cell growth inhibition could be attributed to decreased cell proliferation, impaired cell cycle, and/or increased cell death or apoptosis. To further clarify this issue, colony formation assays were used to assess the cell proliferation ability, while flow cytometry was employed to analyze cell cycle distribution and apoptosis in PHF5A silenced H1299 cells. The results showed that PHF5A depletion inhibited colony formation (Fig. 2f), and resulted in reduced cell populations in both G1 and G2/M phases with significant cell cycle arrest in the S phase (Fig. 3a). Besides, PHF5A suppression also resulted in significantly increased apoptosis (Fig. 3b).

**Fig. 4:**
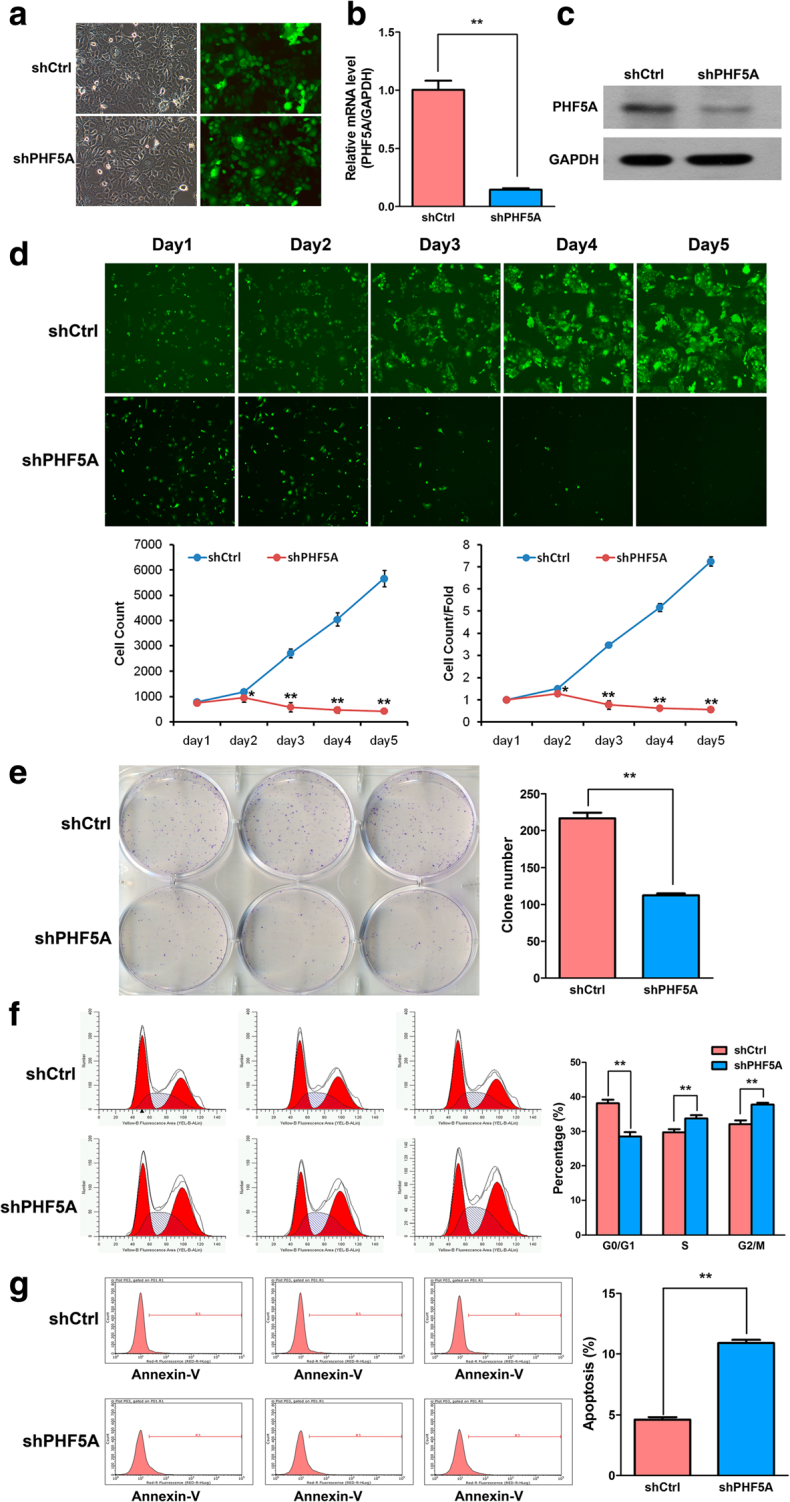
Effect of PHF5A knockdown on cell proliferation, cell cycle, and apoptosis in H1975 cells. **a-c** shRNA against *Phf5a* was conducted by lentivirus infection, and *Phf5a* silencing efficacy in H1975 cells was determined at both mRNA (qRT-PCR) and protein (Western blot) levels. **d** During five days of continuous cell counting by fluorescence microscopy, the quantity of shPHF5A-transfected cells decreased gradually compared with control values. **e** The clonogenic ability of H1975 cells was declined after PHF5A knockdown as evidenced by colony formation assay. **f** Cell cycle analysis using FACS in shCtrl and shPHF5A-transfected H1975 cell lines. **g** Apoptosis was analyzed in PHF5A knockdown and control H1975 cells by FACS after Annexin V staining. ^*^P < 0.05; ^**^P < 0.01

To verify whether the above biological functions of PHF5A were cell-specific, the LAC H1975 cell line, with the highest *Phf5a* expression level among all tested cell lines (Fig. 2a), was also assessed in vitro. Similarly, reduced cell proliferation, impeded colony formation, enhanced S and G2/M phase arrest, and increased apoptosis were observed in PHF5A-silenced H1975 cells (Fig. 4). These results suggested that PHF5A knockdown in LAC cell lines had the general ability to inhibit cell growth in vitro.

### PHF5A knockdown inhibits tumorigenicity in vivo

**Fig. 5:**
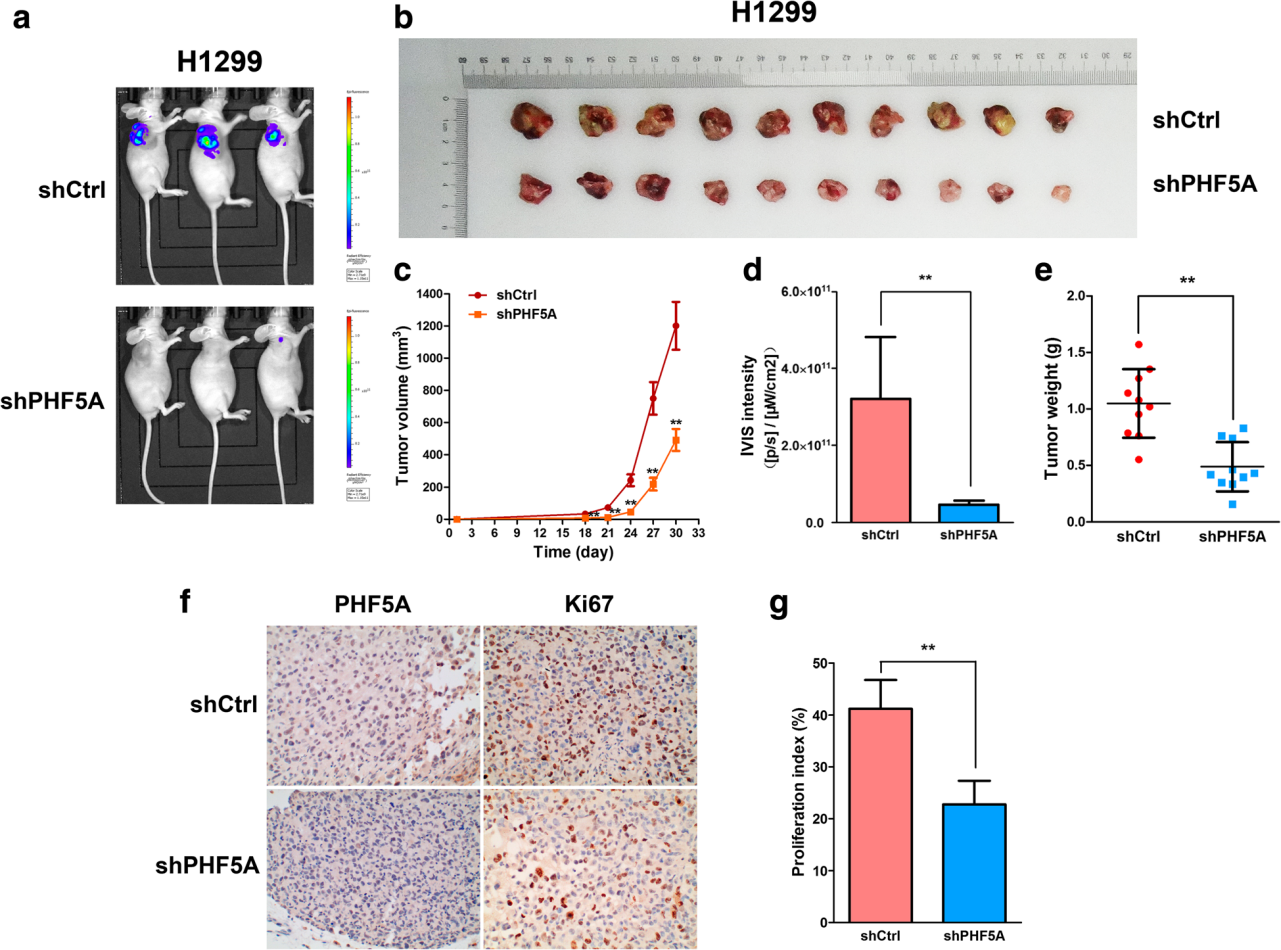
PHF5A silencing inhibits tumor growth in vivo. **a** Representative images of tumor-bearing mice. **b** Xenografts from mice in each group. **c** Tumor volumes were measured at the indicated times. **d** Quantitative IVIS intensity was obtained at 30 days. **e** Mean weights of tumors obtained from mice at 30 days. **f-g** IHC staining demonstrated that PHF5A suppression was established in xenografts, and cell proliferation ability was inhibited in vivo, as indicated by Ki67 levels. ^**^P < 0.01; ^**^compared with the shCtrl group (**c**)

To further assess the oncogenic role of PHF5A in vivo, a xenograft tumor model was established. Nude BALB/c nu/nu mice were subcutaneously injected with H1299-Luc cells transfected with empty or shPHF5A vector. Tumor formation rates in both animal groups were 100%. IVIS spectrum in vivo imaging and intensity analysis demonstrated that mice administered shPHF5A expressing H1299-Luc cells showed significantly reduced luciferase signals at 30 days compared with the control group (Fig. 5a). As shown in Fig. 5b-e, tumor growth, size, and weight were significantly reduced in the PHF5A knockdown group. In addition, IHC analysis confirmed PHF5A downregulation in xenografts collected at 30 days (Fig. 5f), and PHF5A knockdown tumors displayed lower Ki-67 proliferation index compared with controls (Fig. 5f-g). Collectively, these results emphasized the oncogenic role of PHF5A in LAC progression in vivo.

### PHF5A knockdown suppresses invasion and migration in LAC cells in vitro

**Fig. 6:**
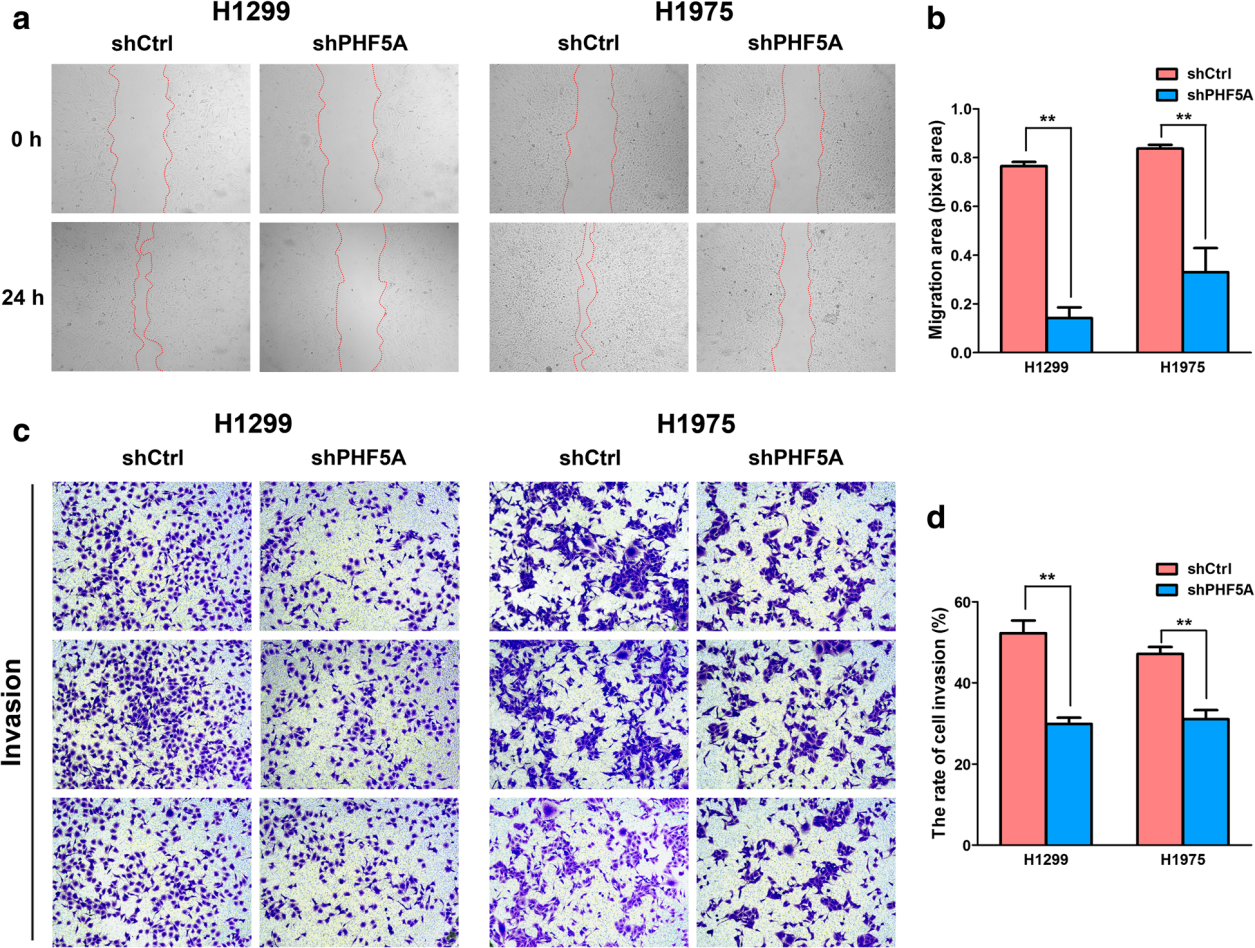
Effect of PHF5A knockdown on migration and invasion in LAC cells. **a** Wound healing assay was performed to assess the migratory potential of control or PHF5A knockdown cells at indicated time points (original magnification, × 100). **b** Quantitation of migration area from control and PHF5A-silenced cells. **c** Transwell invasion assay was carried out to measure the cell invasive ability of control or PHF5A knockdown cells (original magnification, × 100). **d** Quantitation of cell invasion from control and PHF5A-silenced cells. **P < 0.01

Invasion and metastasis are important features of malignant tumors. Since our clinical results and TCGA data consistently indicated that PHF5A expression was not only positively correlated with T stage, but also with lymph node metastasis, we hypothesized that PHF5A could play a role in tumor invasion and migration. Markedly reduced wound closure rates were detected by the wound-healing assay in PHF5A silenced H1299 and H1975 cells, compared with the respective control cells (Fig. 6a and b). Meanwhile, significantly decreased invasive ability was also observed in both cell lines upon PHF5A knockdown as determined by the transwell invasion assay (Fig. 6c and d). These findings strongly indicated that PHF5A was greatly required for invasion and migration in LAC cells.

### PHF5A-regulated downstream genes in LAC cells

**Fig. 7:**
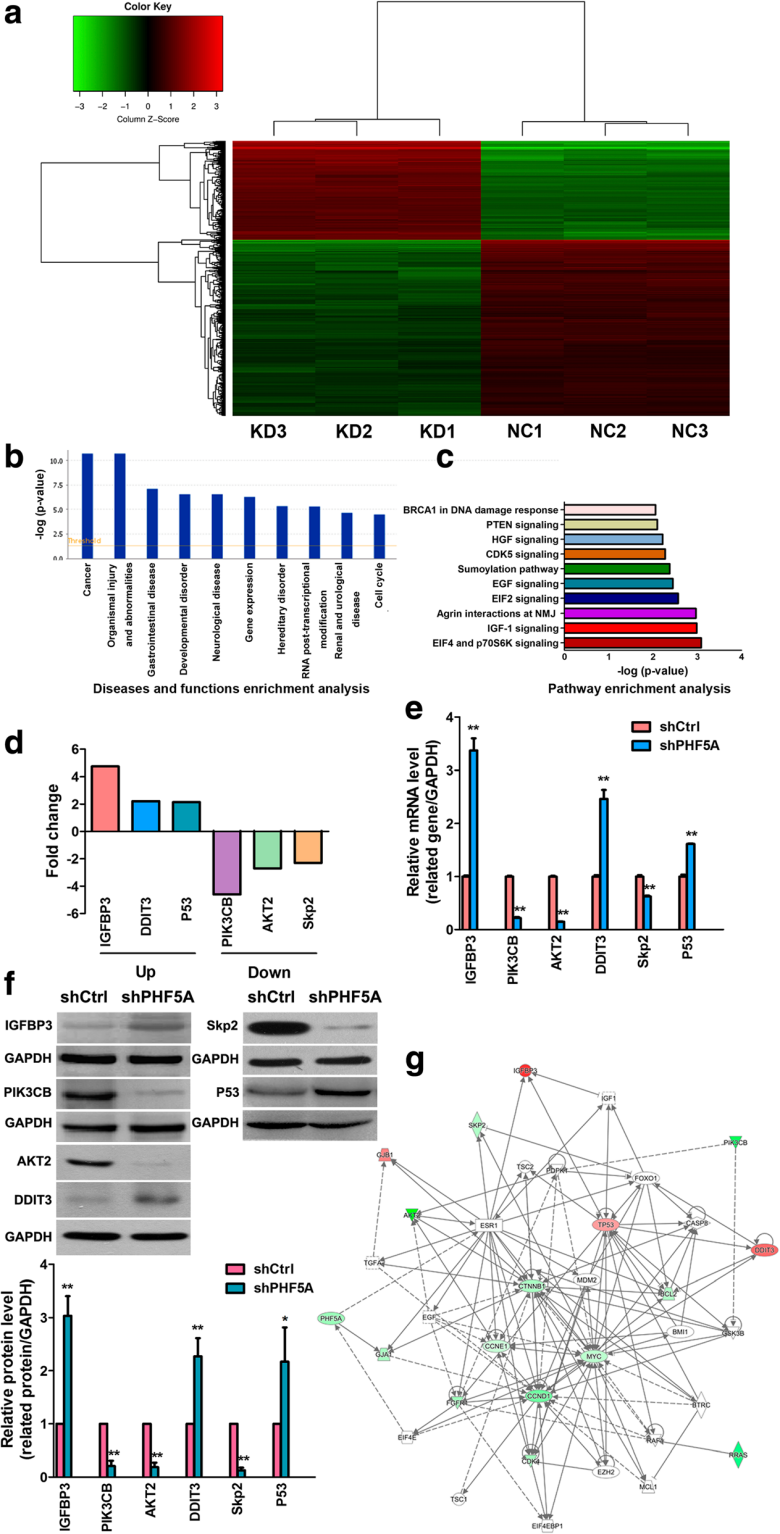
Microarray analysis of H1299 cells after PHF5A knockdown. **a** Hierarchical cluster analysis of shCtrl and shPHF5A-transfected H1299 cells. A total of 1112 genes were differentially expressed. Heat-map colors represent mean-centered fold change expression in log-scale. **b** Disease and function enrichment analysis based on gene numbers, expression levels, and significance probability suggested that among the retrieved categories, genes related to “Cancer” ranked first with the greatest changes. **c** Pathway enrichment analysis for related genes, based on significance probability, suggested that among the analyzed gene sets, genes related to the IGF-1 signaling pathway ranked second in showing the greatest changes in expression levels. **d** Fold changes of three putative IGF-1 pathway genes, including IGFBP3, PIK3CB, and AKT2, as well as cell cycle/apoptosis related genes, including DDIT3, Skp2, and P53, based on microarray analysis. **e-f** Validation of microarray data by qRT-PCR (**e**) and Western blot (**f**). IGFBP3, DDIT3, P53 were significantly up-regulated, while PIK3CB, AKT2, and Skp2 were significantly down-regulated upon PHF5A knockdown. **g** Knowledge-based interactive network for the selected targets in the IGF-1 pathway as well as cell cycle/apoptosis related molecules was constructed with the Reactome database. Red and green denote up-regulated and down-regulated genes, respectively. NMJ, neuromuscular junction (c). ^*^P < 0.05; ^**^P < 0.01, compared with the shCtrl group. A higher quality of Fig. 7 is available as Additional file 6

To elucidate the molecular mechanisms by which PHF5A regulates LAC malignancy, whole genome Affymetrix GeneChip hybridization was applied to assess the gene expression profile after PHF5A knockdown, with IPA performed to identify the potential regulatory pathways involved. Following microarray gene expression and bioinformatics analysis, the shCtrl and shPHF5A groups were clearly distinguished by hierarchical cluster analysis (Fig. 7a). Of the 1112 genes whose expression levels differed by more than 2-fold, 411 and 701 were up-regulated and down-regulated, respectively. Disease and function enrichment analyses indicated marked alterations for each category between control and PHF5A knockdown H1299 cells, and found that “Cancer” ranked first, while “Cell cycle” was included in the significant categories (Fig. 7b). These findings confirmed that PHF5A was closely associated with carcinogenesis, with a potential for cell cycle regulation. Further pathway enrichment analysis demonstrated that besides expression changes in IGF-1 pathway effectors, genes associated with apoptosis and cell cycle pathways were also highlighted (Fig. 7c). The effects of experimental data on IGF-1 pathway related networks as predicted by IPA are depicted in Additional file 5: Figure S2.

According to IPA-based results, key molecules (IGFBP3, PIK3CB, and AKT2) in IGF-1 signaling, as well as representative cell apoptosis/cycle regulatory factors (DDIT3, Skp2 and P53) were assessed for verification. Selected differentially expressed genes from the microarray results were shown in Fig. 7d. qRT-PCR and Western blot demonstrated that the expression levels of IGFBP3, DDIT3, and P53 were significantly increased, while PIK3CB, AKT2, and Skp2 showed decreased amounts, in PHF5A knockdown cells in comparison with control cells (Fig. 7e-f). These findings showed that the expression patterns of these downstream factors were consistent with microarray results after PHF5A depletion. Combining bioinformatics results and subsequent experimental data, the inter-relationships and pathways of PHF5A with cancer associated genes were constructed (Fig. 7g).

## Discussion

The present study demonstrated a novel oncogenic role for PHF5A in lung tumorigenesis and explored the underlying mechanisms. IHC revealed that PHF5A was significantly upregulated in LAC tissues and closely related to tumor progression and poor prognosis in LAC patients. Loss-of-function studies in vitro showed PHF5A knockdown dramatically inhibited LAC cell proliferation and colony formation, induced cell apoptosis, caused cell arrest in the S and (or) G2/M phase, and suppressed migration and invasion abilities. These findings emphasize the role of PHF5A as an oncoprotein in promoting LAC carcinogenesis and progression via multiple signaling pathways.

PHF5A was originally considered a chromatin-associated protein [4, 9]. For example, it was shown that the *Phf5a* gene is expressed ubiquitously in prenatal and postnatal murine tissues, with its encoded protein localized in the nucleus in a non-homogenous pattern [4]. Meanwhile, PHF5A is essential for morphogenetic development in *C. elegans*, with the *Phf5a* gene exhibiting a tissue- and stage-specific pattern of expression [9]. Furthermore, evidence suggests that PHF5A binds to the promoter region of the gene *connexin43*, an important constitute of connexin gene family encoding gap junction protein [19], thereby increasing its expression in response to estrogen induction [5]. Therefore, it was suggested that PHF5A could play a very complex role as a general transcription regulator for different genes. This notion was further supported by characterizing PHF5A as a new subunit of the PHF5A/SAP14b spliceosome associated protein, a component of the pre-mRNA spliceosomal complex splicing factor SF3b [8]. Consequently, as an important component of the RNA processing machinery, PHF5A has been demonstrated to have the ability to interact with splicing factors or alter the splicing process and its coordination of gene expression [7, 8]. Recently, this protein was further found to be essential for the maintenance of pluripotency and cellular reprogramming by directing the transcriptional program [11]; it is also specifically required for normal exon recognition in glioblastoma stem cells to maintain cell expansion and viability [13]. Thus, it was proposed that the PHF5A protein could play a general role in both basic and essential cellular functions, including cancer development.

This study supported the above hypothesis by showing that PHF5A was highly expressed in human LAC tissues, and positively associated with tumor size, lymph node metastasis and clinical stage, and eventually unfavorable prognosis from clinical data. These results were further evidenced by TCGA data. Consistent with these findings, Falck et al. [14] found that *Phf5a* gene expression was significantly increased in endometrial cancer compared with the human benign endometrial tissue, suggesting that expression changes of this gene may be involved in endometrial cancer development. Therefore, we consider that PHF5A may be a potentially highly aggressive and unfavorable prognostic biomarker in LAC. Expectedly, PHF5A knockdown in LAC cells not only decreased tumor growth, but also significantly arrested cell cycle progression, conforming with the defined roles of PHF5A in yeasts described in previous reports [4, 12]. Moreover, we also confirmed a decreased tumor growth in vivo and a suppressed cell invasion and migration capacity in vitro by PHF5A depletion. Thus, we conclude that PHF5A promotes LAC tumorigenesis both in vitro and in vivo.

To explore the possible molecular mechanisms underlying the tumorigenic effects of PHF5A, whole-genome Affymetrix GeneChip analysis was used to screen differentially expressed genes between control and PHF5A-knockdown H1299 cells. Assessment of PHF5A function enrichment was performed with IPA bioinformatics tools; the two classifications of “Cancer” and “Cell cycle” were significant. This was consistent with previous findings [13, 14] and the above experimental data, indicating that PHF5A regulates the cell cycle to participate in tumor development. IGFBP3, PIK3CB, AKT, DDIT3, Skp2, and P53 were subsequently retrieved by the pathway enrichment analysis. At the transcription level, IGFBP3, DDIT3, and P53 were markedly upregulated after PHF5A silencing, while PI3K, AKT2, and Skp2 were downregulated. Subsequently, changes in protein expression levels of these genes were confirmed by Western blot.

Insulin like growth factors (IGFs) constitute an important class of mitogens that activate receptor tyrosine kinases by binding to their receptor IGF-1R, and initiate the downstream PI3K/AKT signaling pathway to induce tumor development [20, 21]. IGFBP3 inhibits the biological effects of IGF-1 by competitive binding to IGF and blocking its downstream signal transduction [22]. In addition, through an IGF-1/IGF-1R-independent pathway, IGFBP3 also regulates cell proliferation, apoptosis, cell cycle, and intracellular metabolism [23, 24]. DDIT3, a transcription factor, is involved in the regulation of multiple genes induced by endoplasmic reticulum stress, e.g. increasing BBC3 and BID expression levels [25, 26] and downregulating the anti-apoptotic factor Bcl-2 [27] to induce apoptosis. The F-box protein family member Skp2 can recognize specific protein substrates such as P21, P27, P53, and cyclins, and controls the degradation of these proteins by ubiquitination, thereby affecting cell cycle progression, cell proliferation, apoptosis, and invasion [28, 29]. Indeed, drugs targeting F-box proteins are promising in the treatment and prevention of human cancers, including lung cancer [29, 30].

PI3K/AKT signaling is not only an indirect downstream effector of the IGF-1/IGFBP3 pathway [20], but also an important upstream regulator of Skp2 [31–33]. It was reported that PI3K/AKT signaling controls the binding of the transcription factor E2F1 to the Skp2 gene promoter and regulates Skp2 at the transcriptional level in pancreatic ductal adenocarcinoma cells [31]. Meantime, Skp2 regulation at both translational and post-translational levels in breast and cervical cancers via this signaling pathway was also observed [32, 33]. Therefore, PHF5A depletion-associated Skp2 down-regulation in the present study might be related to the inactivation of PI3K/AKT2 pathway. Meanwhile, Skp2 downregulation could result in decreased degradation of P53, a target protein for Skp2 ubiquitination, eventually leading to reduced cell proliferation, invasiveness, and tumor progression. Collectively, these results were in line with the prominent role of IGFBP3 as a tumor suppressor [34], and also revealed broad regulatory effects of PHF5A on cellular functions through multiple signaling pathways. Due to the well-characterized role of PHF5A as both a splicing factor and a transcriptional regulator, it is not surprising that the effectors of the pathways affected may undergo different changes at the transcriptional level after PHF5A silencing. Thus, we speculated that the biological effects of PHF5A in LAC cells may represent the combined activities of its downstream effectors resulting from interactions among these signaling pathways.

In conclusion, the current study demonstrated for the first time the important role of the PHD family member and pre-mRNA processing factor PHF5A in LAC tumorigenesis. This function may be related to the regulation of key factors in multiple signaling pathways. The current findings provide new insights into the potential mechanisms underlying the pathogenesis of lung cancer, and may help develop PHD-finger protein inhibitors with promising therapeutic potential.

### Highlights

1. PHF5A overexpression is associated with progression and poor survival in human lung adenocarcinoma (LAC).
2. PHF5A knockdown in LAC suppresses cell proliferation and invasion.
3. PHF5A regulates multiple signaling pathways in LAC, including IGF-1.
4. PHF5A may constitute an oncoprotein and a target for LAC diagnosis and therapy.

## Additional files

Additional file 1: **Table S1.** The primers used for qRT-PCR analysis. (DOC 35 kb)

Additional file 2: **Figure S1.** PHF5A expression in human tissue microarrays containing LAC tissues and adjacent non-tumor lung tissues. The expression of PHF5A protein with yellow or brown staining was predominantly observed in the nucleus of cells in LAC and the normal paired tissues. Representative images of different staining intensities for PHF5A were shown (× 400). (TIFF 18209 kb)

Additional file 3: **Table S2.** Analysis of data from TCGA regarding the relationship between PHF5A expression and lung adenocarcinoma T and N stage. (DOC 39 kb)

Additional file 4: **Table S3.** The correlation between PHF5A expression and T or N stage of human lung adenocarcinoma from data of TCGA. (DOC 30 kb)

Additional file 5: **Figure S2**. Ingenuity pathway analysis identifies protein networks showing inter-relationships and pathways. A sub-network of PHF5A-regulated genes in the IGF-1 pathway was shown. Red and green denote upregulation and downregulation of proteins, respectively. For protein network or pathways analysis, statistical significance was determined by the Fisher’s exact test (*P* < 0.05). (JPEG 1142 kb)

Additional file 6: A higher quality of Fig. 7. (TIF 1760 kb)

## Abbreviations

ANT: Adjacent non-tumor
DDIT3: DNA damage inducible transcript 3
H&E: Hematoxylin and eosin
IGF-1: Insulin-like growth factor-1
IGFBP3: Insulin-like growth factor binding protein 3
IHC: Immunohistochemistry
IPA: Ingenuity pathway analysis
LAC: Lung adenocarcinoma
NC: Negative control
NSCLC: Non-small cell lung cancer
PHD: Plant homeodomain
*Phf5a*: PHD-finger domain protein 5a
PI3K: Phosphatidylinositol 3 kinase
shRNA: Short hairpin RNA
SI: Staining index
Skp2: S-phase kinase associated protein 2
TCGA: The Cancer Genome Atlas
TMA: Tissue microarray
