# Correction: PHD-finger domain protein 5A functions as a novel oncoprotein in lung adenocarcinoma

**DOI:** 10.1186/s13046-026-03787-3

**Published:** 2026-07-23

**Authors:** Yan Yang, Jian Zhu, Tiantian Zhang, Jing Liu, Yumei Li, Yue Zhu, Lingjie Xu, Rui Wang, Fang Su, Yurong Ou, Qiong Wu

**Affiliations:** 1 Department of Medical Oncology, The First Affiliated Hospital of Bengbu Medical College, Bengbu, 233004 People’s Republic of China; 2 Department of Cardiology, The First Affiliated Hospital of Bengbu Medical College, Bengbu, 233004 People’s Republic of China; 3 Department of Pathology, The First Affiliated Hospital of Bengbu Medical College, Bengbu, Anhui 233004 People’s Republic of China

**Correction: J Exp Clin Cancer Res 37, 65 (2018)**

**https://doi.org/10.1186/s13046-018-0736-0**

Following the publication of the original article [1], the authors spotted an error in the figure, specifically:

- Fig. 6a - the image used for shPHF5A-0h group was mistakenly duplicated from the one for shCtrl-0h group

**Incorrect Fig. 6**

**Fig. 6:**
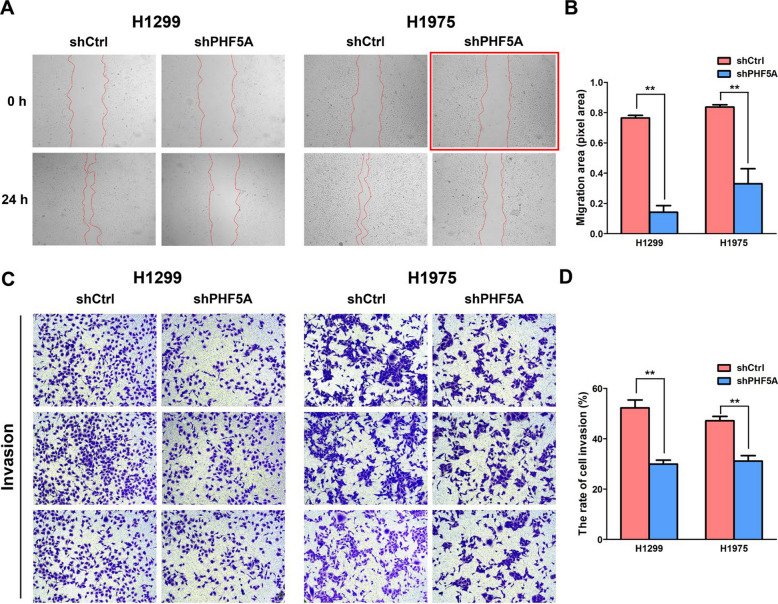
Effect of PHF5A knockdown on migration and invasion in LAC cells. **a** Wound healing assay was performed to assess the migratory potential of control or PHF5A knockdown cells at indicated time points (original magnification, × 100). **b** Quantitation of migration area from control and PHF5A-silenced cells. **c** Transwell invasion assay was carried out to measure the cell invasive ability of control or PHF5A knockdown cells (original magnification, × 100). **d** Quantitation of cell invasion from control and PHF5A-silenced cells. **P < 0.01

**Correct Fig. 6**

**Fig. 6:**
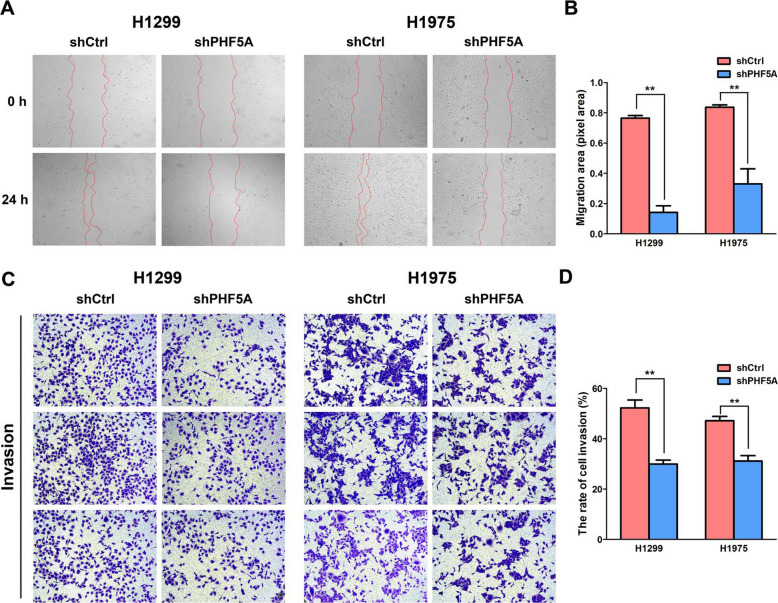
Effect of PHF5A knockdown on migration and invasion in LAC cells. **a** Wound healing assay was performed to assess the migratory potential of control or PHF5A knockdown cells at indicated time points (original magnification, × 100). **b** Quantitation of migration area from control and PHF5A-silenced cells. **c** Transwell invasion assay was carried out to measure the cell invasive ability of control or PHF5A knockdown cells (original magnification, × 100). **d** Quantitation of cell invasion from control and PHF5A-silenced cells. **P < 0.01
