# Supplementary material for: PHD-finger domain protein 5A functions as a novel oncoprotein in lung adenocarcinoma
Source: J Exp Clin Cancer Res. 2018 Mar 22;37:65. doi: 10.1186/s13046-018-0736-0 (PMC5863814; doi:10.1186/s13046-018-0736-0)
Supplement: Supplementary file 1 — Table S1. The primers used for qRT-PCR analysis. (DOC 35 kb) [file 13046_2018_736_MOESM1_ESM.doc]

**Table S1** The primers used for qRT-PCR analysis

| Gene | Sequence | | Product size (bps) |
| --- | --- | --- | --- |
|  | sense（5'-3'） | antisense（5'-3'） |  |
| PHF5A | GTTGCCATCGGAAGACTGT | GCCCCTGGTAAGATCCATAGT | 121 |
| IGFBP3 | CAGAGCACAGATACCCAGAACT | GGACTCAGCACATTGAGGAAC | 119 |
| PIK3CB | TATTTGGACTTTGCGACAAGACT | TCGAACGTACTGGTCTGGATAG | 190 |
| AKT2 | TATACCGCGACATCAAGCTG | GGTCCCACAGAAGGTTTTCA | 125 |
| DDIT3 | GAACCAGGAAACGGAAACAG | ATTCACCATTCGGTCAATCA | 194 |
| Skp2 | AAGAGGAGCCCGACAGTGAGA | GGAGGCACAGACAGGAAAAGAT | 216 |
| P53 | CCTCCTCAGCATCTTATCC | ACAAACACGCACCTCAAA | 258 |
| GAPDH | TGACTTCAACAGCGACACCCA | CACCCTGTTGCTGTAGCCAAA | 121 |
