# Supplementary figures and images for: PHD-finger domain protein 5A functions as a novel oncoprotein in lung adenocarcinoma
Source: J Exp Clin Cancer Res. 2018 Mar 22;37:65. doi: 10.1186/s13046-018-0736-0 (PMC5863814; doi:10.1186/s13046-018-0736-0)

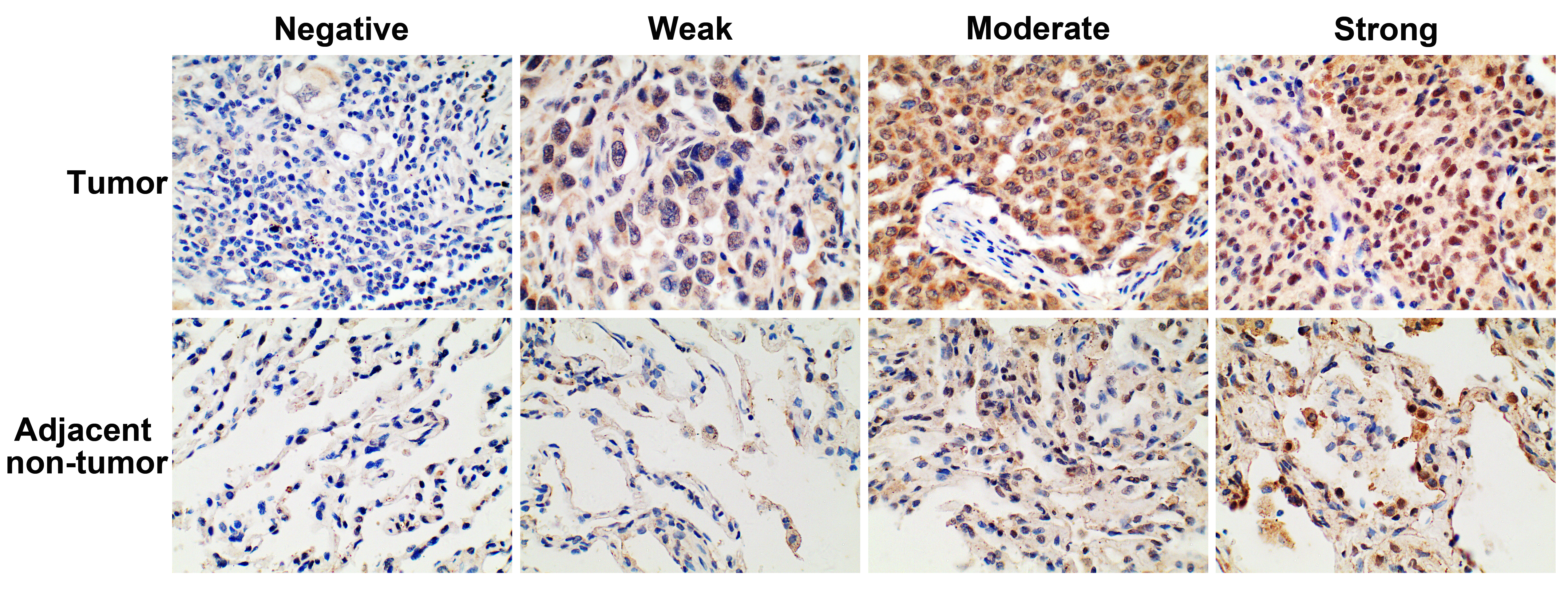

Supplement: Supplementary file 2 — Figure S1. PHF5A expression in human tissue microarrays containing LAC tissues and adjacent non-tumor lung tissues. The expression of PHF5A protein with yellow or brown staining was predominantly observed in the nucleus of cells in LAC and the normal paired tissues. Representative images of different staining intensities for PHF5A were shown (× 400). (TIFF 18209 kb) [file 13046_2018_736_MOESM2_ESM.tif]

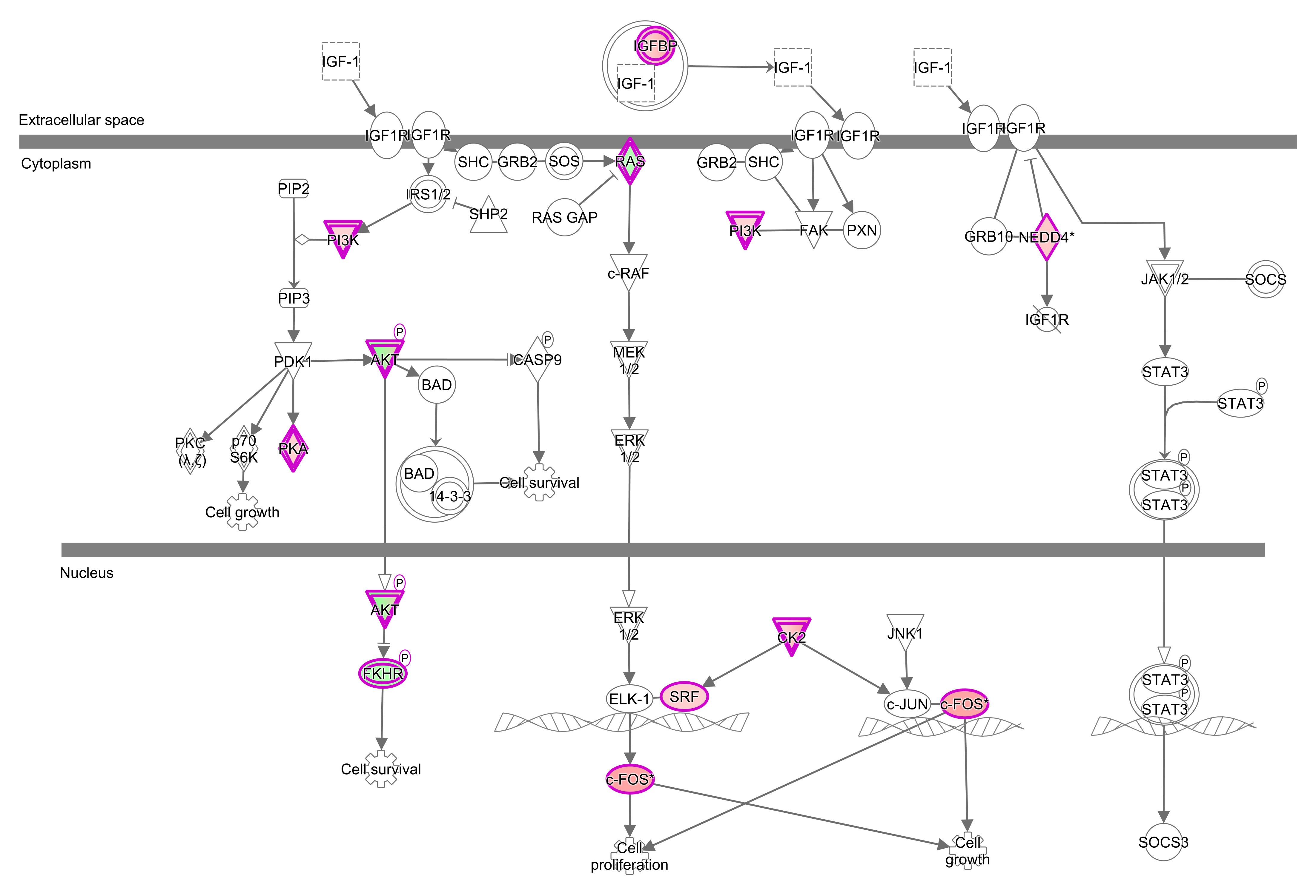

Supplement: Supplementary file 5 — Figure S2. Ingenuity pathway analysis identifies protein networks showing inter-relationships and pathways. A sub-network of PHF5A-regulated genes in the IGF-1 pathway was shown. Red and green denote upregulation and downregulation of proteins, respectively. For protein network or pathways analysis, statistical significance was determined by the Fisher’s exact test (P < 0.05). (JPEG 1142 kb) [file 13046_2018_736_MOESM5_ESM.jpg]

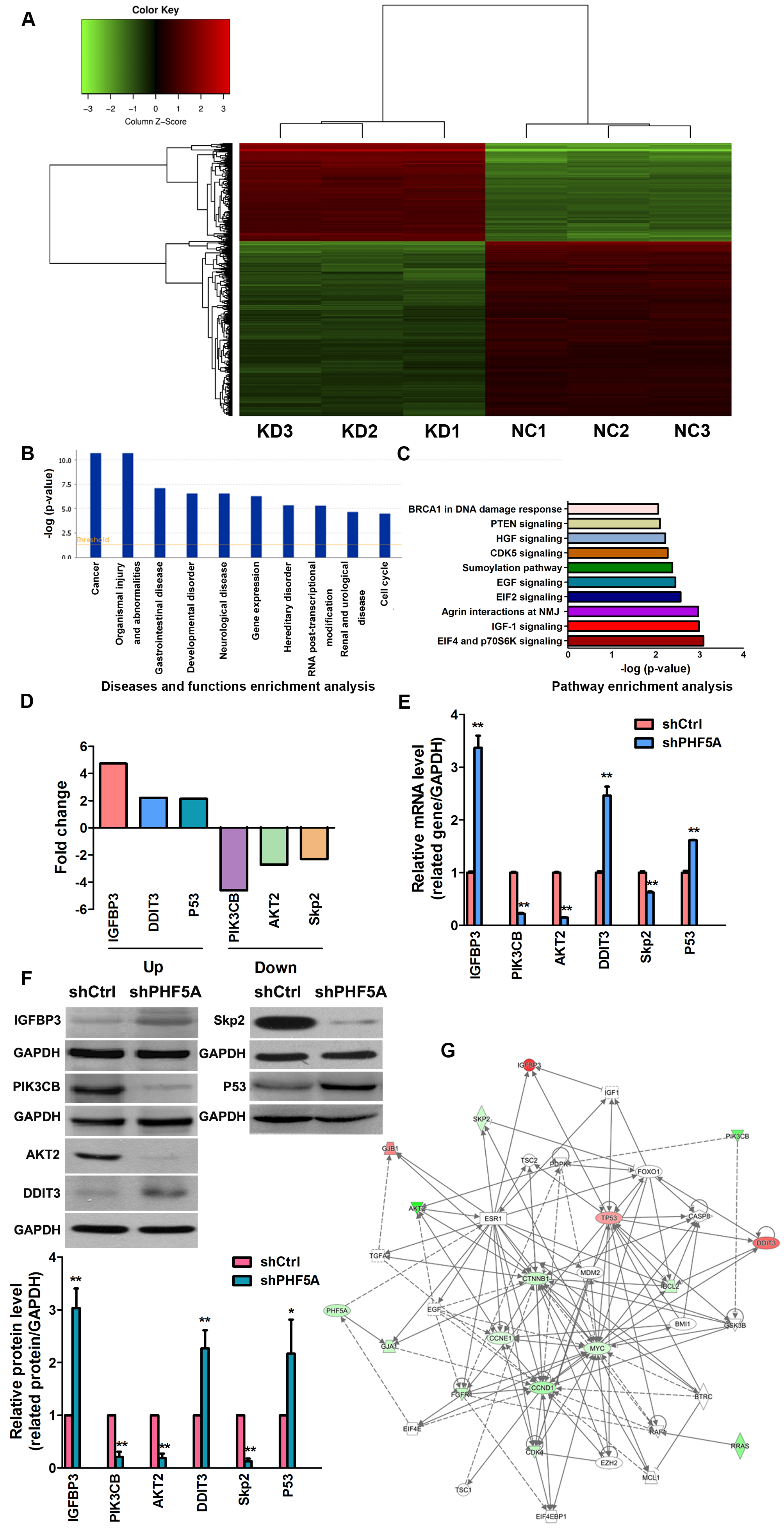

Supplement: Supplementary file 6 — A higher quality of Fig. 7. (TIF 1760 kb) [file 13046_2018_736_MOESM6_ESM.tif]
