# Supplementary material for: PHD-finger domain protein 5A functions as a novel oncoprotein in lung adenocarcinoma
Source: J Exp Clin Cancer Res. 2018 Mar 22;37:65. doi: 10.1186/s13046-018-0736-0 (PMC5863814; doi:10.1186/s13046-018-0736-0)
Supplement: Supplementary file 3 — Table S2. Analysis of data from TCGA regarding the relationship between PHF5A expression and lung adenocarcinoma T and N stage. (DOC 39 kb) [file 13046_2018_736_MOESM3_ESM.doc]

**Table S2** Analysis of data from TCGA regarding the relationship between PHF5A expression and lung adenocarcinoma T and N stage.

|  | | PHF5A expression | | Total (*n*) | *P* value |
| --- | --- | --- | --- | --- | --- |
|  |  | low | high |  |  |
| T stage | T1 | 97 | 68 | 165 | 0.034* |
|  | T2 | 119 | 148 | 267 |  |
|  | T3 | 23 | 22 | 45 |  |
|  | T4 | 9 | 9 | 18 |  |
| Total (*n*) | | 248 | 247 | 495 |  |
| N stage | N0 | 174 | 147 | 321 | 0.014* |
|  | N1/2/3 | 71 | 96 | 167 |  |
| Total (*n*) | | 245 | 243 | 488 |  |

*Significantly different; Mann-Whitney *U* test.
