# Supplementary material for: PHD-finger domain protein 5A functions as a novel oncoprotein in lung adenocarcinoma
Source: J Exp Clin Cancer Res. 2018 Mar 22;37:65. doi: 10.1186/s13046-018-0736-0 (PMC5863814; doi:10.1186/s13046-018-0736-0)
Supplement: Supplementary file 4 — Table S3. The correlation between PHF5A expression and T or N stage of human lung adenocarcinoma from data of TCGA. (DOC 30 kb) [file 13046_2018_736_MOESM4_ESM.doc]

**Table S3** The correlation between PHF5A expression and T or N stage of human lung adenocarcinoma from data of TCGA.

|  |  | **T stage** | | *r* | *P* value | **N stage** | | *r* | *P* value |
| --- | --- | --- | --- | --- | --- | --- | --- | --- | --- |
|  |  | T1 | T2/3/4 |  |  | N0 | N1/2/3 |  |  |
| **PHF5A** | low | 97 | 151 | 0.123 | 0.006* | 174 | 71 | 0.111 | 0.014* |
|  | high | 68 | 179 |  |  | 147 | 96 |  |  |

*Significantly different; Spearman's rank correlation test.
